# Microbiome function predicts amphibian chytridiomycosis disease dynamics

**DOI:** 10.1186/s40168-021-01215-6

**Published:** 2022-03-10

**Authors:** Kieran A. Bates, Ulf Sommer, Kevin P. Hopkins, Jennifer M. G. Shelton, Claudia Wierzbicki, Christopher Sergeant, Benjamin Tapley, Christopher J. Michaels, Dirk S. Schmeller, Adeline Loyau, Jaime Bosch, Mark R. Viant, Xavier A. Harrison, Trenton W. J. Garner, Matthew C. Fisher

**Affiliations:** 1 Department of Zoology, University of Oxford, 11a Mansfield Road, Oxford, OX1 3SZ UK; 2 MRC Centre for GlobaI Infectious Disease Analysis, Department of Infectious Disease Epidemiology, School of Public Health, Imperial College London, London, W2 1PG UK; 3 Institute of Zoology, Zoological Society of London, Regent’s Park, London, NW1 4RY UK; 4 NERC Biomolecular Analysis Facility - Metabolomics Node (NBAF-B), School of Biosciences, University of Birmingham, Birmingham, B15 2TT UK; 5 ZSL London Zoo, Zoological Society of London, Regent’s Park, London, NW1 4RY UK; 6 Laboratoire Écologie Fonctionnelle et Environnement, Université de Toulouse, CNRS, Toulouse INP, Université Toulouse 3 - Paul Sabatier (UPS), Toulouse, France; 7 Department of Experimental Limnology, Leibniz-Institute of Freshwater Ecology and Inland Fisheries (IGB), Alte Fischerhütte 2, 16775 Stechlin, Germany; 8 IMIB Biodiversity Research Institute (CSIC-University of Oviedo), 33600 Mieres, Spain; 9 Biosciences, College of Life and Environmental Sciences, University of Exeter, Exeter, EX4 4DQ UK

**Keywords:** Microbiome, Metabolome, Multi-omics, Amphibian, *Batrachochytrium dendrobatidis*, Chytridiomycosis

## Abstract

**Background:**

The fungal pathogen *Batrachochytrium dendrobatidis* (*Bd*) threatens amphibian biodiversity and ecosystem stability worldwide. Amphibian skin microbial community structure has been linked to the clinical outcome of *Bd* infections, yet its overall functional importance is poorly understood.

**Methods:**

Microbiome taxonomic and functional profiles were assessed using high-throughput bacterial 16S rRNA and fungal ITS2 gene sequencing, bacterial shotgun metagenomics and skin mucosal metabolomics. We sampled 56 wild midwife toads (*Alytes obstetricans*) from montane populations exhibiting *Bd* epizootic or enzootic disease dynamics. In addition, to assess whether disease-specific microbiome profiles were linked to microbe-mediated protection or *Bd*-induced perturbation, we performed a laboratory *Bd* challenge experiment whereby 40 young adult *A. obstetricans* were exposed to *Bd* or a control sham infection. We measured temporal changes in the microbiome as well as functional profiles of *Bd*-exposed and control animals at peak infection.

**Results:**

Microbiome community structure and function differed in wild populations based on infection history and in experimental control versus *Bd-*exposed animals. *Bd* exposure in the laboratory resulted in dynamic changes in microbiome community structure and functional differences, with infection clearance in all but one infected animal. *Sphingobacterium*, *Stenotrophomonas* and an unclassified Commamonadaceae were associated with wild epizootic dynamics and also had reduced abundance in laboratory *Bd*-exposed animals that cleared infection, indicating a negative association with *Bd* resistance. This was further supported by microbe-metabolite integration which identified functionally relevant taxa driving disease outcome, of which *Sphingobacterium* and *Bd* were most influential in wild epizootic dynamics. The strong correlation between microbial taxonomic community composition and skin metabolome in the laboratory and field is inconsistent with microbial functional redundancy, indicating that differences in microbial taxonomy drive functional variation. Shotgun metagenomic analyses support these findings, with similar disease-associated patterns in beta diversity. Analysis of differentially abundant bacterial genes and pathways indicated that bacterial environmental sensing and *Bd* resource competition are likely to be important in driving infection outcomes.

**Conclusions:**

*Bd* infection drives altered microbiome taxonomic and functional profiles across laboratory and field environments. Our application of multi-omics analyses in experimental and field settings robustly predicts *Bd* disease dynamics and identifies novel candidate biomarkers of infection.

Video Abstract

**Supplementary Information:**

The online version contains supplementary material available at 10.1186/s40168-021-01215-6.

## Introduction

Amphibians are one of the most vulnerable animal lineages on the planet with over 40% of species threatened with extinction [1]. The pathogenic chytrid fungi *Batrachochytrium dendrobatidis* (*Bd*) and *B. salamandrivoran*s (*Bsal*) are the causative agents of the skin disease chytridiomycosis [2, 3] which is a major driver of global amphibian declines. To date, chytridiomycosis has decimated over 500 amphibian species, representing the greatest loss of biodiversity due to a pathogen ever recorded [4]. Understanding the determinants of chytridiomycosis outbreak dynamics in the wild is therefore necessary to not only control disease spread and mitigate ecosystem-level impacts [5] but also elucidate the disease ecology of these super-generalist pathogens.

Amphibian skin and its microbiota are the first line of defence against pathogenic invaders [6, 7] including *Bd* [8, 9]. Crucially, microbes typically have short generation times, high rates of mutation and large within-host population sizes that facilitate faster adaptation of defensive traits than is possible in long-lived hosts [10]. Host-associated microbes may provide protection against infection by outcompeting pathogens for resources [11], producing antimicrobial compounds [12–14], priming the host immune system [15] and in some instances preying upon pathogens [16]. Prior studies have identified bacterial-derived antimicrobial compounds that inhibit *Bd* growth [12–14, 17, 18]; however, determining overall microbiome community function remains challenging due to the complex web of interactions occurring amongst microbes [17, 19]. Microbial taxa can also switch from being inhibitory to promoting pathogen growth based on community composition [19] and in more extreme cases can shift from being beneficial to parasitic (or vice-versa) in processes that may be mediated by host factors [20, 21], the abiotic environment [22] or microbial community ecology [23]. As well as their role in defence, microbial communities are important in maintaining host dermal homeostasis [24–27]. Disruption to microbial community function and microbe-host co-metabolic pathways (often driven by pathogen invasion) can consequently negatively impact host health [28–32]. Microbiome function therefore cannot be predicted by taxonomy alone and studies need to integrate functional inter-relationships amongst microbes and their hosts.

We combine omics methods to determine how amphibian skin microbial community structure and function shape chytridiomycosis disease dynamics in a wild outbreak and laboratory in vivo infection model. Our wild system centres on midwife toad (*Alytes obstetricans*) populations in the French and Spanish Pyrenean mountains that present epizootic or enzootic disease dynamics based on long-term observations of sustained *Bd-*induced population decline or recovery respectively [33]. A prior study of the Pyrenean system demonstrated no clear link between pathogen genetics and epidemiological trends, but a strong association between bacterial community structure and disease dynamics [33]. The functional relevance of these bacterial community differences with relation to disease is however not known.

We show that cross-kingdom microbial communities are associated with *Bd* infection dynamics in the wild and that *Bd* clearance in the laboratory is associated with an altered microbiome state. Further, microbiome function maps to taxonomic beta diversity in both the laboratory and field, exhibiting a disease-associated profile. These findings indicate that taxa differences predict differences in function and are therefore not consistent with functional redundancy.

## Methods

### Ethics statement

Fieldwork was performed under licence from the Parc National des Pyrénées (2016-110 and 111) and the Instituto Aragonés de Gestión Ambiental. Animal experiments were carried out in accordance with The Animals (Scientific Procedures) Act of 1986 Directive 2010/63/EU and followed all codes of practice which reinforce this law.

### Field sampling

This study focussed on four *Alytes obstetricans* populations in the French and Spanish Pyrenees (SI Fig. 1, SI Table 1) that are part of a long-term *Bd* surveillance project [33]. In recent years, three populations (Ibon Acherito, Puits d’Arious and Lhurs) have exhibited low *Bd* infection intensity and stable population abundance consistent with the development of *Bd* resistance and enzootic disease dynamics. Conversely, Arlet is typified by epizootic disease dynamics based on continued population decline since the outbreak of *Bd* in 2005, high *Bd* infection loads and perennially high mortality rates [33]. We sampled fourteen *A. obstetricans* metamorphs for *Bd*, skin microbes and metabolome from each population (*n* = 56) in August 2016. New nitrile gloves were worn for each animal. Animals were rinsed with sterile water to remove transient microbes/substrate debris; then, microbial communities and *Bd* were each sampled using a single sterile MW100 cotton dry tipped swab (MWE Medical Wire, Corsham, UK) that was rolled over the ventral and dorsal surfaces of the skin ten times, and hindlimbs five times. To collect skin mucosal metabolome, each animal was placed in a falcon tube containing 12 ml of sterile water for 20 min to allow metabolites to diffuse into solution. Metabolites were collected using solid phase extraction (Supplementary methods).

### Experimental infection

Forty adult *A. obstetricans* bred in captivity from wild-caught animals from Pyrenean populations (Arlet, Ansabere and Lhurs) were randomly assigned to 1.6L plastic enclosures containing an autoclaved damp paper towel and a plastic cover object and were fed crickets (*Acheta domesticus*) ad libitum twice weekly. Animals were housed individually for two weeks prior to the experiment to allow acclimatisation to experimental conditions. Enclosures were cleaned twice weekly with Rely+ on Virkon (Antect International Ltd., Suffolk, UK). The experimental facility was kept on a 12h light/dark cycle and maintained at 18°C. Animals were swabbed prior to *Bd* exposure on day 1 and on days 30 and 60 post-exposure. Twenty animals were repeatedly exposed on days 1, 3, 5, 7, 9 and 11 to 2 ml of 1.5 × 10^4^ *Bd* zoospores for 4 h with the remaining 20 control animals being exposed to a sham infection containing only nutrient media (mTGHL). On day 30, metabolome samples were collected from each animal following the same protocol as field samples. On day 60, all animals were euthanised.

### Sample processing/analysis

Microbial genomic DNA was extracted from swabs using the DNeasy Blood and Tissue kit (Qiagen, Venlo, Netherlands) according to the manufacturer’s instructions. A mutanolysin pre-treatment was included to enhance bacterial DNA recovery [34]. We amplified the V4 region of the 16S rRNA gene and the ITS2 region of the fungal internal transcribed spacer (ITS) using custom barcoded primers and PCR conditions adapted from a prior study [35] (Supplementary methods). 16S and ITS2 data was analysed using DADA2 [36] and our previously published method [31] respectively (Supplementary methods).

*Bd* DNA was extracted using a bead-beating protocol [37]. Extractions were diluted 1/10 before undergoing qPCR amplification with samples run in duplicate [37] and with *Bd* standards of 100, 10, 1 and 0.1 zoospore genomic equivalents (GE). Samples with greater than 0.1 GE were considered *Bd* positive.

Metabolome samples were analysed in a randomised order by ultra-high-pressure liquid chromatography-mass spectrometry (UHPLC-MS) (Supplementary methods).

Shotgun metagenome sequencing was performed using an Illumina HiSeq 2000 on a subset of samples from the field study (*n* = 50) and day 30 of the experiment (*n* = 20). DNA sequences were merged, quality checked and annotated using the Metagenomics Rapid Annotation (MG-RAST) server (vs. 4.0.3.) with default parameters [38]. Taxonomic analysis was performed using the RefSeq database with the following cut-off parameters: *e*-value of 1E-5, minimum identity of 60%, maximum alignment length of 15 bp and constrained to only bacterial reads. Functional analyses of bacteria were performed using the KEGG subsystem database.

### *Bd* infection analysis

*Bd* genomic equivalents data was log_10_+1 transformed to fit a Gaussian distribution and analysed using ANOVA. A Tukey’s post-hoc test was performed to identify statistically significant differences between populations.

### Alpha diversity

Bacterial reads per sample for the laboratory and field study had a mean of 27011 (sd = 10448) and 22830 (sd = 10378) respectively. Fungal sequences per sample for the field and laboratory study had a mean of 731 (sd = 1183) and 2570 (sd = 5668) respectively. To mitigate the effects of uneven sampling [39] bacterial field and laboratory samples were rarefied to 3658 and 10692 sequences respectively, corresponding to the depth of the lowest read samples. Fungal samples for the field study were rarefied to 233 reads per sample resulting in 35 samples out of 56 of the sequenced samples remaining for downstream analysis. For the laboratory challenge experiment, fungal samples were rarefied to 200 reads per sample resulting in 84 samples remaining. To examine whether bacterial and fungal Shannon diversity differed across populations, we performed ANOVA and Tukey’s post-hoc test. For the 16S data, Shannon diversity was log transformed to improve fit to a Gaussian distribution. For our experiment, we performed a non-parametric Mann-Whitney test for each time point to compare Shannon diversity in the control and *Bd*-exposed treatment.

Shotgun metagenomics functional KO (KEGG Orthology) data ranged from 2101 to 1,344,436 KO reads per sample for the field data and 812,729 to 2,410,428 KO reads per sample for the laboratory data. We rarefied our data to 9847 and 812,729 KO reads for our field (leaving 47 samples) and laboratory data respectively. Differences in Shannon diversity between populations in the field and *Bd* exposure in the laboratory were assessed using ANOVA and a *t*-test respectively.

### Beta diversity and differential abundance

Fungal taxonomic data was filtered to remove samples with fewer than 233 and 200 sequences for the field and laboratory studies respectively. For shotgun metagenomic field data, we filtered samples with fewer than 9000 KO reads resulting in 47 samples remaining. Bacterial 16S and shotgun data were subsequently filtered to include features with a mean relative abundance > 0.01% and fungal data was filtered to include taxa with a relative abundance > 0.05%. Zero values attributed to rare taxa/features were replaced using the Bayesian “CZM” method using the zCompositions R package [40] before centred-log ratio (CLR) transformation. To determine if beta diversity of 16S, ITS2, shotgun and metabolome data differed based on population and disease state in the wild, or *Bd* treatment in the laboratory, each dataset was visualised using principal components analysis (PCA) using mixomics [41]. To quantify differences in beta diversity based on host population and disease dynamic (wild) or day, *Bd* and their interaction (laboratory), we applied permutational multivariate analysis of variance (PERMANOVA) on Euclidean distances using the adonis function in the R package vegan [42] with 999 permutations.

To identify ASVs/OTUs/KOs driving differences in beta diversity based on wild disease dynamic or on experimental treatment on day 30 (the time point corresponding to peak *Bd* infection), we performed sparse partial least squares-discriminant analysis (sPLS-DA) [43] using mixomics [41]. We tuned our sPLS-DA models (function tune.splsda, mixomics package) using 10 × 5-fold cross-validation to determine optimal model parameters (number of components and feature selection). The performance of our sPLS-DA models were assessed using 100 × 5-fold cross-validation. We further identified ASVs/OTUs/KOs that differed in abundance in the epizootic and enzootic populations or experimental treatment for days 1, 30 and 60 of the experiment using ALDEx2 [44] and selected informative features based on Benjamini-Hochberg (BH) corrected Welch’s *t* test *p*-values < 0.05 and an effect size 1> and < − 1.

To assess the congruency of taxonomic data generated from shotgun metagenomics and the bacterial taxonomic 16S data in the field, we performed Procrustes analysis on Euclidean distances of RefSeq CLR-transformed abundance and CLR-transformed 16S data using the R package vegan [42]. The PROTEST function was used which performs repeated symmetric analyses to estimate if the degree of association of the two matrices is greater than that expected by chance alone.

Wilcoxon’s tests were performed to determine differentially abundant metabolites based on disease dynamic (wild) or *Bd* exposure (laboratory). *P*-values were adjusted using the false discovery rate procedure and significant features that also had a log2 fold change > 1.5 were considered most informative as possible biomarkers. Partial least squares discriminant analysis (PLS-DA) was performed using mixomics [41]. The PLS-DA model was assessed using 5-fold cross-validation repeated 50 times and model significance was tested using a permutation analysis (999) implemented using MVA.test in the RVAideMemoire package [45]. Three components were selected for PLS-DA for field data (NMC = 0.02, *p* = 0.001) and laboratory data (NMC = 0.078, *p* = 0.001). Metabolites driving differences in metabolome variation based on wild disease dynamic or laboratory pathogen exposure were identified based on the variable influence on the projection (VIP) parameter on component 1. The most informative metabolites were determined based on a VIP score > 2 and that also showed a statistically significant profile at *q* < 0.05 and log_2_ fold change > 1.5 from the univariate analysis.

### Omics integration

To determine the degree of association between bacterial/fungal taxonomic community structure and overall metabolite composition, we performed Procrustes analysis on Euclidean distance matrices using the vegan package [42]. To determine if a multi-omics signature could differentiate enzootic/epizootic disease dynamics or laboratory *Bd* exposure on day 30 for bacteria and metabolite data, we applied the supervised method, data integration analysis for biomarker discovery using latent components (DIABLO) [46]. We used the CLR-transformed microbiome data (filtered to the top 0.01% of bacterial ASVs) and g-log-transformed metabolite data as inputs. The block link was set to 0.1. Model parameterisation (perf function) was performed to select the number of components to use in our final models. A tuning procedure (tune.block.splsda) with 50 × 10-fold cross-validation was applied to determine the optimal number of variates. Final model performance was assessed using 10-fold cross-validation repeated 50 times. We did not apply supervised analysis to our fungal data due to poor model fit.

To cross-validate our supervised analysis and also examine fungi-metabolite associations in the field, we applied an unsupervised omics integration method-sparse partial least squares (sPLS) regression, using the mixomics package in R [41]. For model parameterisation, we set the number of metabolite features to include in our model for each component (keepY) to 111, corresponding to the number of metabolites found to be discriminative of disease dynamic based on single omics analyses. We selected 21 bacterial ASVs to be included for each component (keepX), which corresponds to the number of differentially abundant taxa with an effect size > 1 or < − 1 and corrected *p*-values < 0.05 as identified from ALDEx2 analysis based on disease dynamic. For wild fungal-metabolite interactions, we used a keepX of 10 given the low number of differentially abundant fungal OTUs. We visualised microbe-metabolite associations with a correlation co-efficient > 0.65 using relevance networks plotted in cytoscape [47].

We report shared features recovered from single omics and DIABLO analyses for the laboratory experiment and field study. We identified metabolite features with identical KEGG annotations and therefore are more likely to be the same metabolite. For bacterial taxa identified as discriminatory in the laboratory and field studies, we performed Spearman’s correlation of CLR-transformed taxa abundance and log_10_+1 GE load for day 30 experimental data.

## Results

### Wild *Bd* infection dynamics

**Fig. 1:**
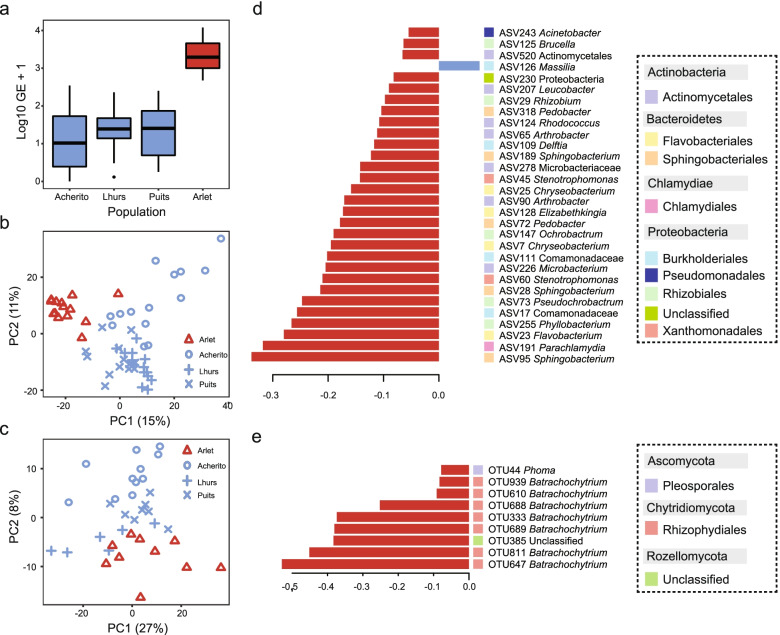
Metagenomic sequencing-based exploration of *Bd* disease dynamics supports functional differences in skin bacterial communities from epizootic and enzootic populations. PCA and PERMANOVA of bacterial KO beta diversity for **a**) all KOs **b**) Metabolism (KEGG level 1) **c**) Environmental Information Processing (KEGG level 1) **d**) Cellular Processes (KEGG level 1). **e **Clustered image map of bacterial KOs (**annotated by functional pathway**) contributing to separation along sPLS-DA component 1. Samples are clustered using complete linkage and Euclidean distances. Sample sizes: Acherito *n* = 12, Lhurs *n* = 11, Puits *n* = 10, Arlet *n* = 14

*Bd* load was lower in all enzootic populations relative to the epizootic (ANOVA F_(3, 52)_ = 33.65, *p* < 0.001, SI Table 2, Fig. 1a). *Bd* prevalence was 100% in all populations except Ibon Acherito (79%).

### Host skin bacterial and fungal taxonomic composition is associated with disease dynamic and population

Bacterial Shannon diversity differed based on population (ANOVA F_(3, 52)_ = 8.58, *p* < 0.001), with Acherito being significantly higher than Arlet (*p* < 0.001) and Lhurs (*p* < 0.001). Fungal Shannon diversity did not differ by population *(p* > 0.05*).* Disease dynamic and population varied in beta diversity of bacteria and fungi (Bacteria: disease dynamic PERMANOVA Pseudo-F_(1, 54)_ = 7.08, *R* ^2^ = 0.12, *p* = 0.001, population PERMANOVA Pseudo-F_(3, 52)_ = 6.04, *R* ^2^ = 0.26, *p* = 0.001; Fungi: disease dynamic PERMANOVA Pseudo-F_(1, 33)_ = 2.89, *R* ^2^ = 0.08, *p* = 0.002, population PERAMANOVA Pseudo-F_(3, 31)_ = 2.79, *R* ^2^ = 0.21, *p* = 0.001, Fig. 1b, c).

Twenty-nine bacterial ASVs were associated with epizootic dynamics and a single ASV was associated with enzootic populations from sPLS-DA (Fig. 1d, Supplementary Data 1). Nine fungal OTUs were associated with epizootic dynamics on sPLS-DA component 1 (Fig. 1e), whilst component two had 22 and 8 OTUs linked to enzootic and epizootic dynamics respectively (Supplementary Data 2). Seven out of nine fungal OTUs on component 1 were classified as *Batrachochytrium* (Fig. 1e), whilst OTUs on component 2 belonged to eight classified fungal classes (Supplementary Data 2)*.* Differential abundance analysis using ALDEx2 identified 21 bacterial biomarkers for disease state, of which 1 and 20 were associated with enzootic and epizootic disease dynamics respectively (Supplementary Data 3). Discriminatory bacterial taxa spanned four phyla and seven classes. For the enzootic populations, the single discriminatory ASV (also identified using sPLS-DA) was assigned to the *Massilia* genus (order: Burkholderiales), whilst ASVs in the epizootic population belonged to the Sphingobacteriales, Actinomycetales, Burkholderiales, Chlamydiales, Flavobacteriales, Rhizobiales and Xanthomonadales. ALDEx2 identified one fungal OTU (*Batrachochytrium* sp.) significantly associated with epizootic dynamics (Supplementary Data 4).

### Host bacterial community function associates with disease dynamic and population

**Fig. 2:**
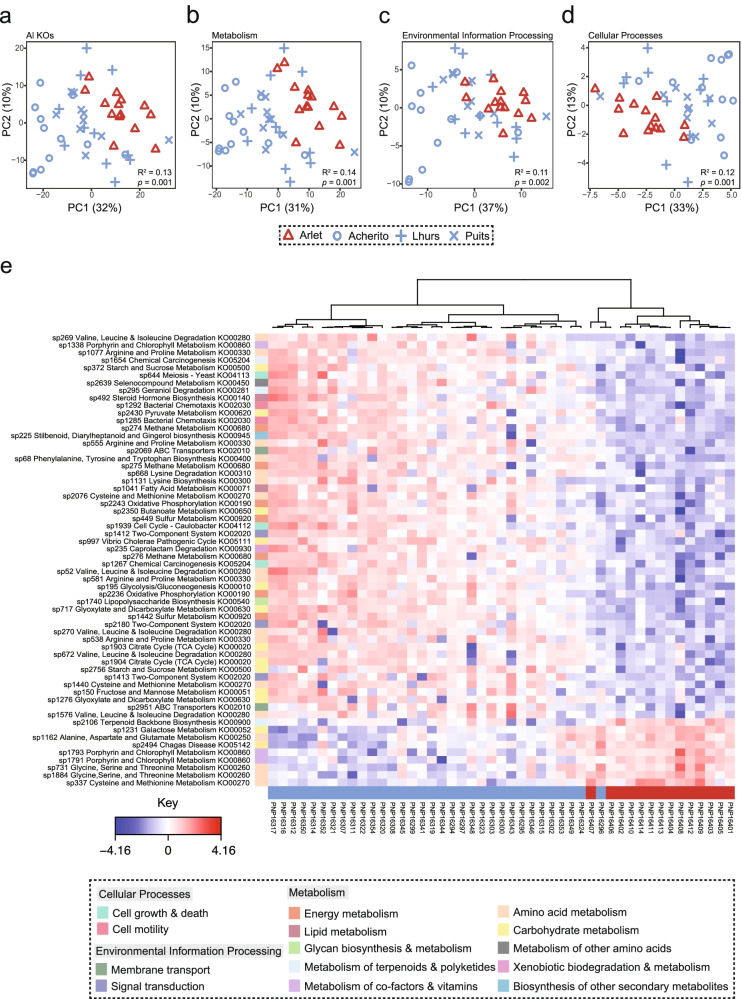
*Bd* infection alters functional profile of the amphibian skin bacterial microbiome. **a **PCA of bacterial KO abundance on day 30 of the *Bd* exposure experiment **b**) PCA of Metabolism (KEGG level 1) **c**) PCA of environmental processes (KEGG level 1) **d**) Clustered image map of bacterial KOs (annotated by functional pathay) associated with *Bd* or control exposure as identified by sPLS-DA. Sample sizes: control group *n* = 11, *Bd*-exposed group *n* = 9

Procrustes analysis showed significant correlation between shotgun bacterial taxonomic data and 16S data (Procrustes m12 squared = 0.43, correlation in symmetric Procrustes rotation = 0.76, *p* = 0.001). Shannon diversity of KO hits differed based on population (ANOVA F_(3, 43)_ = 9.56, *p* < 0.001) with Acherito being significantly higher than Arlet (*p* < 0.001) and Lhurs (*p* < 0.001), whilst Puits was also higher than Arlet (*p* = 0.03). KO beta diversity differed based on disease dynamic and population (disease dynamic PERMANOVA Pseudo-F_(1, 45)_ = 6.80, *R* ^2^ = 0.13, *p* = 0.001, population PERMANOVA Pseudo-F_(3, 43)_ = 4.50, *R* ^2^ = 0.24, *p* = 0.001, Fig. 2a–d). Analysis of bacterial KOs using sPLS-DA found the main sources of variation was in amino acid metabolism, carbohydrate metabolism and energy metabolism (Fig. 2e, Supplementary Data 5). ALDEx2 identified 72 KOs, of which 17 and 55 were associated with epizootic and enzootic populations respectively (Supplementary Data 6).

### Host skin metabolome maps to disease dynamic and population

**Fig. 3:**
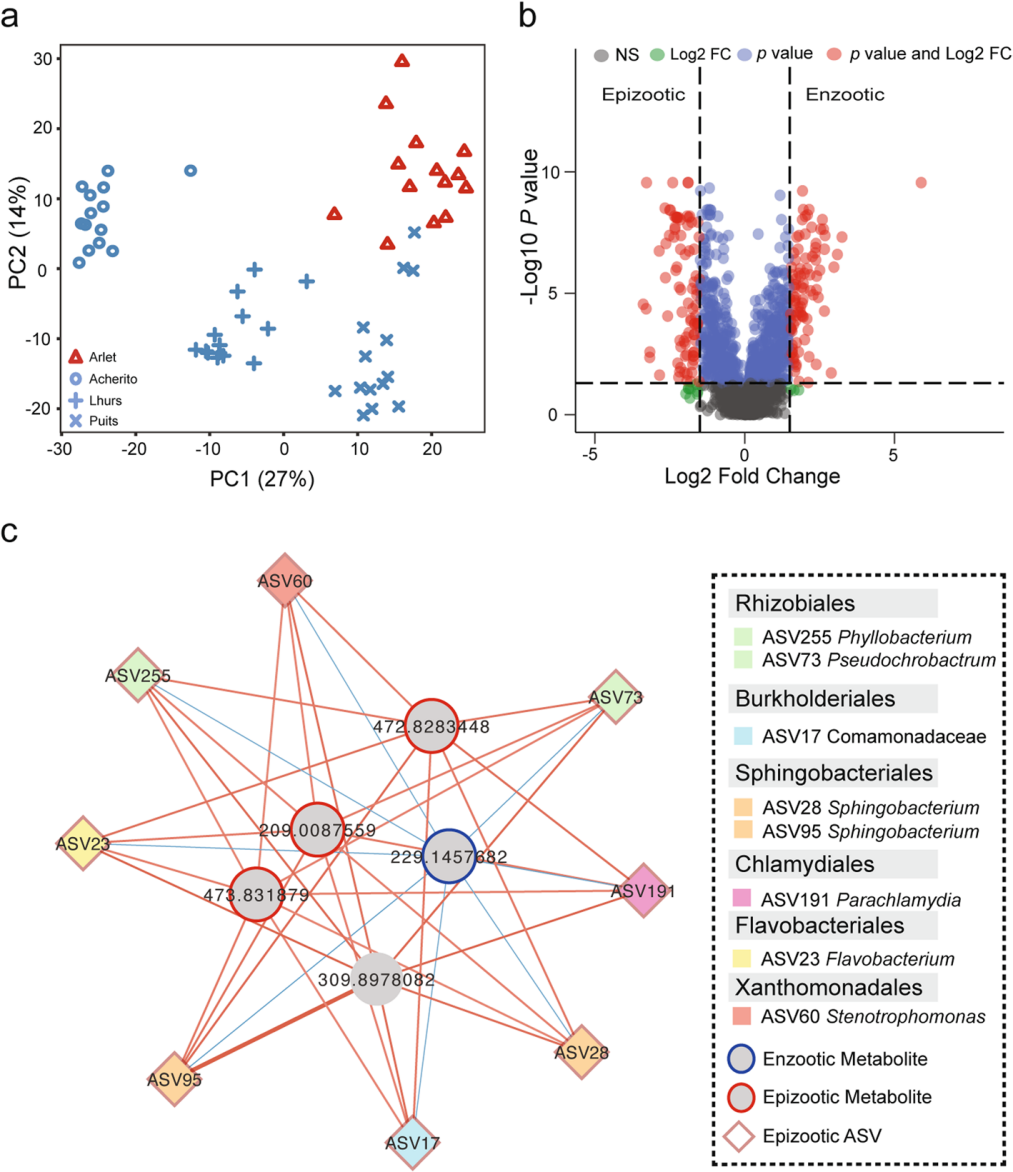
Multi-omics integration selects predictive targets of wild disease dynamics. **a**) PCA of skin metabolite profile of wild *A. obstetricans* populations **b**) volcano plot displaying differentially abundant metabolite features identified by univariate Wilcoxon’s test. **c**) Relevance networks produced by integration of microbiome and metabolome datasets using DIABLO for bacteria-metabolite interactions. A single network was identified that was indicative of epizootic dynamics based on the presence of taxa that were identified as enriched in the epizootic population from single omics analyses and their positive associations with epizootic metabolites. Bacteria are shown as diamonds and metabolites as circles. A positive correlation between nodes is indicated by red connecting lines, a negative correlation is shown by blue. Enzootic and epizootic enriched metabolites/bacteria have blue and red borders respectively. Sample sizes: Acherito *n* = 14, Lhurs *n* = 14, Puits *n* = 14, Arlet *n* = 14

2014 metabolite features were measured in negative ion mode. Disease dynamic and population were significant factors driving variability in metabolome (PERMANOVA dynamic Pseudo-F_(1, 54)_ = 10.93, *R* ^2^ = 0.17 *p* = 0.001, population Pseudo-F_(3, 52)_ = 13.14, *R* ^2^ = 0.43, *p* = 0.001, Fig. 3a). Univariate analysis identified 870 differentially abundant metabolite features, of which 183 had a log_2_ fold change > 1.5 (Fig. 3b, Supplementary Data 7). PLS-DA based on disease dynamic yielded 121 metabolite features with a VIP score > 2, of which 111 were considered most informative (VIP > 2, *q* < 0.05, log_2_ fold change > 1.5, Supplementary Data 8). Following putative annotation of the most informative metabolite features, one of the features was suspected to be the anti-*Bd* metabolite indole-3-carboxaldehyde. More detailed investigation of the MS/MS data for this metabolite (*m/z* 144.04606 RT 6.96 min) kindled our interest in a second, following the peak of the same *m/z* in the spectra at 8.73 min, present in the XCMS matrix but originally filtered out as thought to be due to some higher blank signal. However, upon closer inspection, the intensities of this second peak behaved similarly to *m/z* 144.04606 with higher intensity in the epizootic population (Wilcoxon’s test *W* = 17, *p* < 0.001, SI Fig. 2). Whilst both ions were putatively annotated as the anti-*Bd* metabolite indole-3-carboxaldehyde, the MS/MS data for the second signal resembled those in spectral databases (https://mona.fiehnlab.ucdavis.edu/spectra/display/PR100508; https://mona.fiehnlab.ucdavis.edu/spectra/display/PB000507) and from an authentic standard fragmented in-house, thus providing evidence for the presence (and increased abundance in the epizootic population) of indole-3-carboxaldehyde on wild *A. obstetricans* skin.

### Microbiome/metabolome interactions in the wild

Procrustes analysis showed a significant association between bacterial/fungal taxonomy and metabolic profile (bacteria: m12 square = 0.45, correlation in symmetric Procrustes rotation = 0.74, *p* = 0.001; fungi: m12 square = 0.60, correlation in symmetric Procrustes rotation = 0.63, *p* = 0.004). DIABLO confirmed enrichment of eight bacterial ASVs in the epizootic population that were also identified from a single omics analysis. Of the five informative metabolites recovered from our analysis, three were indicators of epizootic disease dynamics and one was linked to enzootic dynamics (Wilcoxon’s test *q* < 0.05, log2 fold change > 1.5, Supplementary Data 9). Network analysis identified a single cluster representative of epizootic dynamics based on the presence of epizootic discriminatory taxa and their positive correlations with the epizootic enriched metabolites (Fig. 3c). Meanwhile, negative correlations were found between epizootic taxa and the single metabolite feature that was enriched in the enzootic populations.

Unsupervised sPLS regression supports findings from supervised analysis with 31 of the 40 bacteria-metabolite pairs from DIABLO also recovered in sPLS. We identified 450 correlated pairs of bacterial ASVs/metabolite features (39 negative, 411 positive) involving 20 ASVs and 157 metabolite features forming two sub-networks (SI Fig 3a,b, Supplementary Data 10). Bacterial sub-network A was reflective of epizootic dynamics based on a dominance of epizootic discriminative ASVs that were positively correlated with epizootic enriched metabolite features. *Sphingobacterium* had the greatest betweenness centrality (a measure of how often a node occurs on all shortest paths between two other nodes and therefore an indicator of functional importance). In bacterial sub-network B, an ASV belonging to the Sinobacteraceae family and *Haliscomenobacter* exhibited the greatest betweenness centrality.

Fungal-metabolome analysis also yielded two sub-networks comprising 461 fungal-metabolite pairs (152 negative, 309 positive) involving 11 fungal OTUs and 109 metabolite features (SI Fig 4a,b, Supplementary Data 11). Fungal sub-network A comprised 8 OTUs, all of which were discriminant for epizootic dynamics from sPLS-DA, with ALDEx2 also identifying one OTU (sp633) as discriminant for epizootic dynamics. Fungal sub-network A was dominated by chytrid with 7 OTUs classified as *Batrachochytrium* sp. Twenty-seven metabolite features in fungal sub-network A were positively correlated with fungal taxa that were discriminative for epizootic dynamics, whilst twenty-three metabolite features were negatively correlated with fungal taxa in subnetwork A that were discriminative for enzootic dynamics. Fungal sub-network B comprised three OTUs—two belonging to the Massarinaceae and an unknown Dothidiomycete.

### Experimental infection alters skin microbiome state

Nineteen out of twenty *Bd*-exposed animals became infected with *Bd* by day 30, with *Bd* infection intensity ranging from 0.88 to 427.08 genomic equivalents (GE) (SI Fig 5). On day 60, a single *Bd*-exposed animal remained infected, with all other animals clearing infection. There was no mortality.

**Fig. 4:**
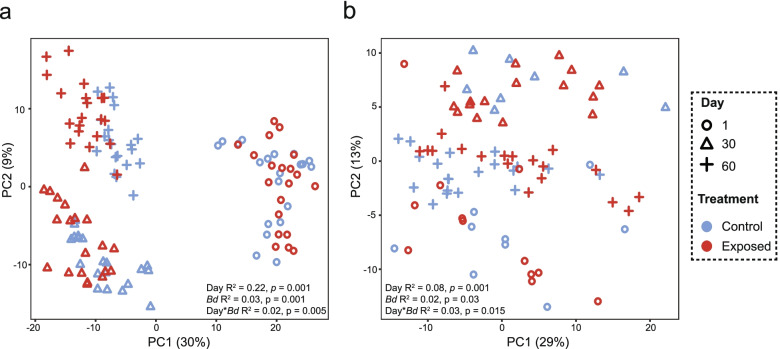
Experimental *Bd* infection perturbs host skin bacterial and fungal communities. Beta diversity of **a**) bacteria and **b**) fungi during experimental *Bd* infection*.* Sample sizes bacteria: control = 20 (each sample day), *Bd* exposed = 20 (each sample day). Sample sizes fungi: day 1 control = 9, day 1 exposed = 12, day 30 exposed = 16, day 30 control = 8, day 60 exposed = 19, day 60 control = 20

We show no effect of *Bd* exposure on bacterial Shannon diversity at any time point measured (*p* > 0.05). Fungal Shannon diversity was reduced in *Bd*-exposed animals compared to the control group on day 30 of the experiment (*W* = 109, *p* = 0.005) but not on day 0 or 60 (*p*>0.05). We confirm no significant difference in beta diversity for bacteria and fungi at the start of the experiment (PERMANOVA *p* > 0.05). Beta diversity differed by day, *Bd* exposure and their interaction for bacteria (PERMANOVA day F_(1,116)_ = 34.24, *R* ^2^ = 0.22, *p* = 0.001, *Bd* F_(1,116)_ = 4.92, *R* ^2^ = 0.03, *p* = 0.001, day*Bd F_(1,116)_ = 3.15, *R* ^2^ = 0.02, *p* = 0.005) and fungi (PERMANOVA day F_(1,80)_ = 7.52, *R* ^2^ = 0.08, *p* = 0.001, *Bd* F_(1,80)_ = 2.05, *R* ^2^ = 0.02, *p* = 0.034, Day*Bd F_(1,80)_ = 2.30, *R* ^2^ = 0.03, *p* = 0.015, Fig. 4a, b). Twenty bacterial ASVs and two fungal OTUs were found to drive differences in beta diversity on day 30 according to sPLS-DA (Supplementary Data 12, 13). The bacterial ASVs spanned three phyla and eight classes, whilst fungal OTUs all belonged to the Basidiomycete phylum and the Class Tremellomycetes. ALDEx2 found no differentially abundant bacterial or fungal taxa at day 0. At day 30, three bacteria and one fungal taxa differed between treatments (Supplementary Data 14, 15). *Sphingobacterium* and *Acinetobacter* were associated with the control group, whilst *Comomonas* was increased in the *Bd* exposure group. The fungus *Trichosporon* had significantly higher abundance in the control group compared to *Bd*-exposed animals on day 30. For day 60, we identified three discriminatory taxa (*Comamonas*, *Brevundimonas* and *Gemmobacter*, Supplementary Data 16), all of which were associated with *Bd* exposure. No fungal taxa were differentially abundant at day 60.

**Fig. 5:**
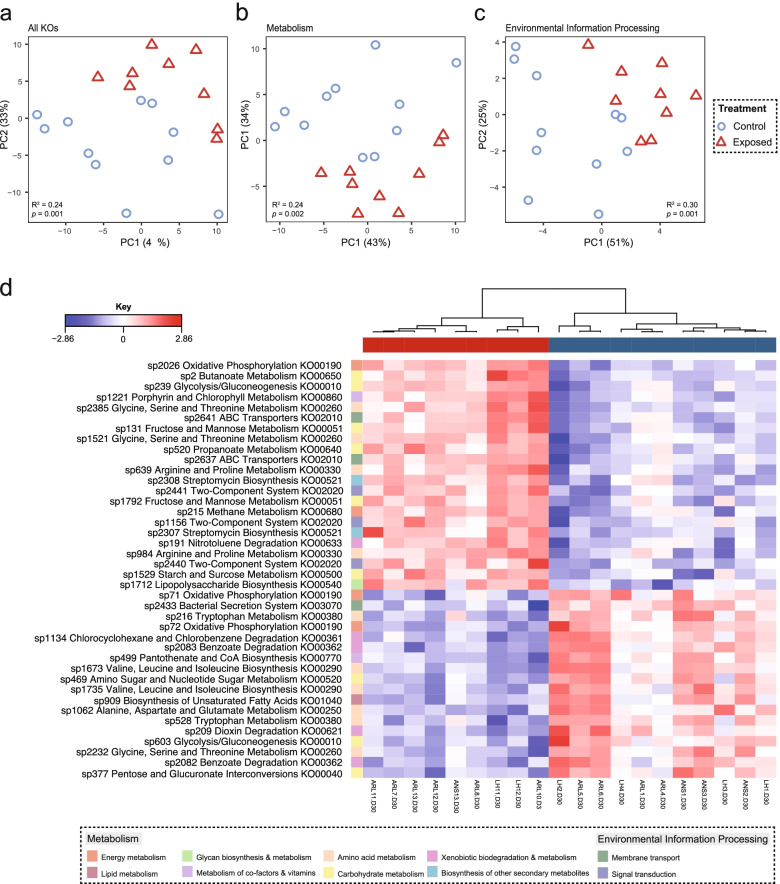
*Bd* infection alters functional profile of the amphibian skin bacterial microbiome. **a** PCA of bacterial KO gene abundance on day 30 of the *Bd* exposure experiment **b**) PCA of Metabolism (KEGG level 1) **c**) PCA of environmental processes (KEGG level 1) **d**) Clustered image map of bacterial KO genes associated with *Bd* or control exposure as identified by sPLS-DA. Sample sizes: control group *n* = 11, *Bd*-exposed group *n* = 9

KO Shannon diversity at day 30 was lower in the *Bd*-exposed group than the control (*t*-test df = 17.43, *t* = 2.96, *p* = 0.009). KO beta diversity differed by *Bd* exposure (PERMANOVA F_(1, 18)_ = 5.69, *R* ^2^ = 0.24, *p* = 0.001, Fig. 5a–c). Forty discriminatory KOs were identified from sPLS-DA (Fig. 5d, Supplementary Data 17). ALDEx2 identified 303 discriminatory KOs, with 114 and 189 increased in the control and *Bd*-exposed groups respectively (Supplementary Data 18). Differentially abundant KO hits were mostly associated with amino acid metabolism, carbohydrate metabolism, membrane transport, signal transduction and metabolism of cofactors and vitamins.

UHPLC-MS metabolomics resulted in a peak list with 2329 features. Metabolome differed based on *Bd* exposure (PERMANOVA F_(1, 38)_ = 4.50, *R* ^2^ = 0.11, *p* = 0.003). Wilcoxon’s tests identified 729 features of which 37 had a log_2_ fold change > 1.5 and *q* < 0.05 (Supplementary Data 19). PLS-DA produced separation based on *Bd* exposure with 111 metabolite features having a VIP score > 2, of which 24 had a log_2_ fold change > 1.5 and *q* < 0.05 (Supplementary Data 20). Screening for anti-*Bd* metabolites found an increased abundance in the *Bd*-exposed group of a metabolite feature putatively annotated as indole-3-carboxaldehyde (*q* < 0.05).

**Fig. 6:**
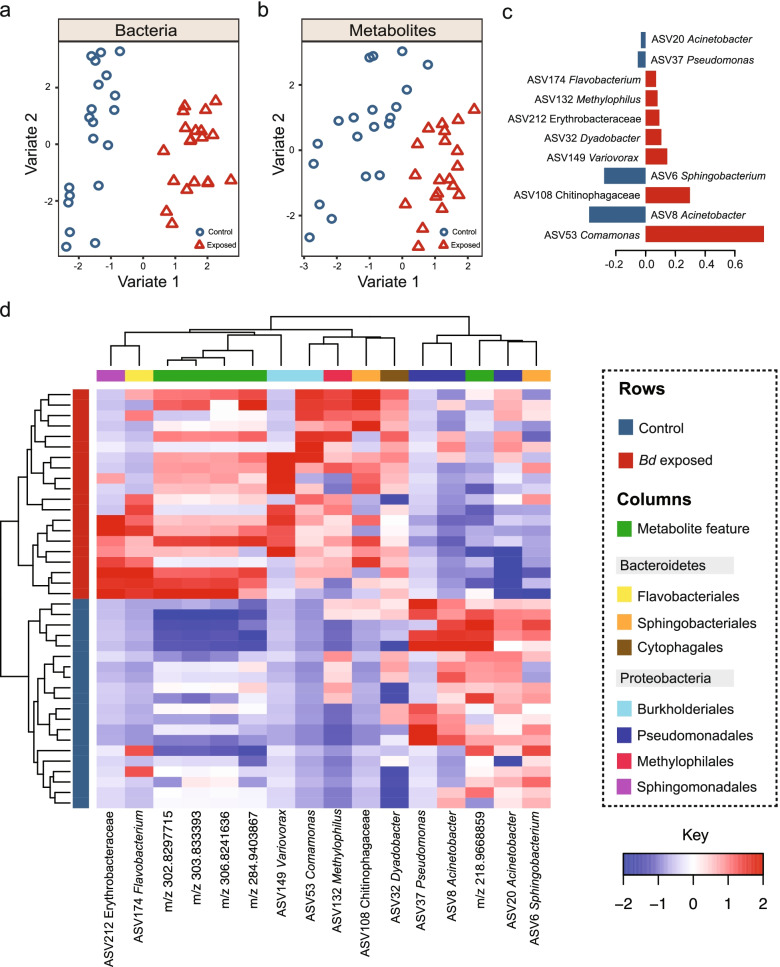
Integration of skin bacterial microbiome and metabolome identifies a *Bd* infection-associated multi-omics signature. DIABLO sample plots demonstrating discrimination of *Bd*-exposed and un-exposed midwife toads based on **a**) skin bacterial microbiome and **b**) skin metabolome **c**) bacterial taxa contributing separation along component 1 in **(a)**. Bar length indicates loading coefficient weight of selected bacterial ASVs. Bar colour indicates the group in which the bacterial ASV has the highest median abundance, blue = control, red = *Bd* exposed. **d** Clustered image map (Euclidean distance, complete linkage) of the multi-omics signature. Samples are represented in rows, selected features of the first component are shown in columns. Sample sizes: Control = 20, *Bd* exposed = 20

Microbiome/metabolome integration using DIABLO [48] identified bacterial and metabolite features discriminating control and exposure treatments (Fig. 6a,b, Supplementary Data 21). *Bd* exposure was associated with enrichment of bacterial ASVs and metabolite features (Fig. 6c, d) indicating that infection drives changes in microbe-metabolite interactions. All three discriminatory bacterial ASVs (*Acinetobacter*, *Sphingobacterium*, *Comamonas*) identified using ALDEx2 were also identified by DIABLO.

### Biomarkers in the laboratory and field

We identified common biomarkers in the laboratory and field for bacterial 16S, shotgun and metabolome, but not fungal ITS2 data (Supplementary Data 22). Three bacterial ASVs (*Sphingobacterium*, *Stenotrophomonas*, Comamonadaceae) were discriminatory in the laboratory and field, with increased abundance in the epizootic population but reduced abundance in the *Bd*-exposed experimental group (in which all animals survived), indicating an association with negative health outcomes. Of these taxa, *Stenotrophomonas* abundance was significantly positively correlated with *Bd* infection intensity on day 30 of the experiment (SI Fig 6). Eighteen bacterial KOs were discriminatory in both the laboratory and field. Six metabolite annotations from the laboratory and field were identical including indole-3-carboxaldehyde, a bacterial-derived anti-*Bd* metabolite [12] that was increased in the epizootic population and in the *Bd*-exposed laboratory treatment.

## Discussion

We show that bacterial and fungal taxonomy, bacterial functional profile and skin metabolome reflect *Bd* disease dynamics in the wild and exposure in the laboratory. *Batrachochytrium* clearance coinciding with an altered microbiome state in laboratory animals supports prior findings [30, 31, 48], but here suggests a non-detrimental or even protective microbiome akin to wild enzootic populations. Conversely, the distinct microbiome community structure and function of the epizootic population suggests a deleterious relationship with amphibian health in the context of infection [30, 31].

The existence of a detrimental microbiome profile with epizootic dynamics is further supported by the high relative abundance of taxa with known positive associations with *Bd*, or that have been associated with co-infections in amphibians. Of note are *Sphingobacterium*, *Rhodococcus*, *Parachlamydia*, *Chryseobacterium*, *Flavobacterium*, *Acinetobacter* and *Stenotrophomonas*, which were identified as indicator taxa of epizootic dynamics in the previous year [33] and are known to contribute to amphibian ill health or promote *Bd* growth [30, 31, 49–53]. Three bacterial ASVs (*Sphingobacterium*, *Stenotrophomonas* and an unclassified Commamonadaceae) were discriminatory for epizootic dynamics in the field but associated with the control group of the experiment, indicating that high abundance of these taxa is linked with poor infection outcomes. Spearman’s correlation revealed a positive relationship between taxa abundance and *Bd* infection at day 30 of the experiment for *Stenotrophomonas* (significant) and *Sphingobacterium* (non-significant), further indicating a synergistic relationship between these taxa and *Bd*.

*Batrachochytrium* sp. were also the dominant fungal taxa associated with epizootic disease dynamics, indicating that high *Bd* infection load is associated with microbiome differences. A single bacterium identified as *Massilia* had increased abundance in the enzootic population and is known to be *Bd* inhibitory in the laboratory [52], producing the *Bd* inhibitory metabolite violacein [54]. This finding suggests that bacterial taxa other than *Janthinobacterium lividum* reside on the amphibian skin and may confer similar protective benefits, further highlighting the importance of considering bacterial function as well as taxonomy. Given the opposite patterns of discriminate taxa abundance in animals that cleared infection in the laboratory and from the wild epizootic population, our findings suggest that *Bd* resistance is driven by stemming proliferation of certain detrimental bacteria coinciding with *Bd* colonisation and that protection may therefore be better predicted by broader aspects of community ecology.

Skin microbiome function also differed based on *Bd* exposure/disease dynamics, as demonstrated by disease-associated patterns in beta diversity for shotgun metagenomic and metabolomic profiles. Specifically, we found disease-associated differences in bacterial metabolism of amino acids, carbohydrates, glycans, terpenoids and polyketides, vitamins and cofactors and energy metabolism. We also found differences in bacterial environmental information processing suggesting that *Bd* infection results in changes to both the metabolic environment and bacterial interactions.

Bacterial gene abundance in wild enzootic populations and laboratory *Bd*-exposed animals supports possible microbe-mediated protection through enrichment of genes of potential importance in bacteria-*Bd* competition. For example, arginine decarboxylase and ornithine decarboxylase cause a dose-dependent reduction of *Bd* growth up to 84% when blocked in vitro [55] and were increased in *Bd*-exposed laboratory animals and wild enzootic populations respectively. In laboratory *Bd*-exposed animals, we also find evidence for zinc competition through increased abundance of the bacterial genes znuA (involved in environmental zinc uptake) and superoxide dismutase (SOD1), which uses a zinc cofactor*.* Bacterial competition for zinc provides a novel theoretical mechanism of *Bd* growth limitation and virulence attenuation, since zinc is required by *Bd* virulence factors (m36 metalloproteases) [56, 57]. An increased abundance of bacterial chitin deacetylase, which breaks down the fungal cell wall component chitin [58] into the antifungal agent chitosan [59] was also found in laboratory *Bd*-exposed animals and may be a bacterial mechanism of *Bd* inhibition.

Bacterial environmental information processing and sensing was identified as a key factor differentiating *Bd* infection/disease dynamics. Specifically, genes involved in the bacterial two-component system (TCS) exhibited increased abundance in the enzootic populations and the *Bd*-exposed treatment in the laboratory. The TCS plays an important role in bacterial sensing and adaptation to environmental stimuli [60] where it can modulate bacterial virulence [61], antimicrobial resistance [62], environmental stress [63, 64], biofilm production [65] and is a key component in the establishment of symbiosis [66, 67]. In our laboratory experiment, we identified a large number of differentially abundant genes from the ATP-binding cassette (ABC) transporter family, which can be regulated by the TCS [68]. Differential abundance of TCS and ABC transporters with *Bd* infection is likely a response to changes in the skin molecular environment through both host and *Bd* derived metabolites. Of particular importance may be TCS-mediated resistance to antimicrobial peptides (AMPs) via ABCs transporters [69]. *Alytes obstetricans* is well known for its production of AMPs and toxins by skin serous glands [70]. Therefore, living in concert with this antimicrobial arsenal is critical to the survival of beneficial amphibian skin-associated bacteria in order to colonise, persist and work mutualistically with host defences. We find evidence for this in the laboratory *Bd*-exposed treatment, which showed increased abundance of genes involved in the yejABEF microcin C transporter system that confers resistance to AMPs in other host models [71].

Putative bacterial-produced *Bd* inhibitory metabolites were also found in both the laboratory and field, with the anti-*Bd* compound indole-3-carboxaldehyde discovered as a biomarker for laboratory *Bd* exposure and wild epizootic dynamics. *Janthinobacterium* (an indole-3-carboxaldehyde producer) [13] was also present in both the laboratory and field (although did not significantly differ in abundance) as were bacterial genes from the tryptophan pathway (in which indole-3-carboxaldehyde is a metabolic product). The increased abundance of putative indole-3-carboxaldehyde in the epizootic population may indicate that anti-*Bd* metabolites alone are not enough to counter infection and that indole-3-carboxaldehyde production may be *Bd* dose dependent.

We found strong correlations between microbial taxonomy and skin metabolome. This likely reflects host-derived metabolites shaping microbiome structure and microbe-derived metabolites contributing to the skin metabolome. Microbiome and metabolome integration recovered discriminatory taxa and metabolite features that were also identified from single omics analyses, providing confidence in their classification as disease biomarkers. *Stenotrophomonas* and *Sphingobacterium* are of particular interest given their positive association with epizootic dynamics in the wild, but negative association with *Bd* clearance in the laboratory (Supplementary Data 22). The high betweenness centrality of *Sphingobacterium* in the epizootic bacteria-metabolite network supports its importance in driving differences in microbiome function based on disease. *Batrachochytrium* was identified as the most common fungal taxa associated with metabolome phenotype and also had the greatest betweenness centrality in the wild epizootic population, indicative of *Bd*-driven metabolic perturbation, as previously shown in moribund frogs [72]. Links between multiple microbial taxa and single metabolite features may resemble functional redundancy within a population, whereby multiple taxa utilise/produce the same metabolite feature [73]. Alternatively, metabolite production may be contingent on interactions between microbial taxa, such as the anti-*Bd* metabolite tryptophol, which is produced in bacterial co-culture but not in monoculture [17]. Associations between microbial taxa and individual metabolites may also be important for microbiome community assembly, if the presence of one taxon is dependent on the production of a metabolite by another, for example through cross-feeding mechanisms [74]. Investigating microbe-metabolite interactions further will therefore be valuable in enhancing our understanding of the ecological processes governing the amphibian skin microbiome and its role in disease.

## Conclusion

We applied 16S and ITS2 high-throughput amplicon sequencing, shotgun metagenomics and metabolomics to establish the relationship between amphibian skin microbiome taxonomic profile and community function. We demonstrate that bacterial and fungal taxonomic community composition strongly predicts skin metabolome profile, indicating a lack of functional redundancy. Further, we show that *Bd* drives changes in microbiome community structure, bacterial gene abundance and skin metabolome during laboratory infection and in a wild outbreak. Our integration of complex biological data and identification of shared multi-omics features in the laboratory and field provide mechanistic insight into the role of the microbiome in *Bd* infection outcome. These results lay the foundations for future studies examining microbiome-*Bd* interactions and will be valuable towards developing biomarkers as early warning beacons of epizootics in nature.

## Supplementary Information

**Additional file 1.** Supplementary Information.

**Additional file 2.** Supplementary data.

**Additional file 3.** Supplementary Methods.

## Data Availability

16s and ITS2 Sequence data have been deposited on the BioProject database under accession codes PRJNA777609 and PRJNA778055 respectively. Shotgun data for the laboratory and field can be accessed at https://www.mg-rast.org/linkin.cgi?project=mgp100552 and https://www.mg-rast.org/linkin.cgi?project=mgp91053 respectively. Other data are available upon request from the authors.

## Abbreviations

ASV: Amplicon sequence variant
*Bd*: *Batrachochytrium dendrobatidis*
CLR: CentredLog-Ratio
DIABLO: Data integration analysis for biomarker discovery using latent components
KEGG: Kyoto Encyclopaedia of Genes and Genomes
OTU: Operational taxonomic unit
PCA: Principal components analysis
PERMANOVA: Permutational multivariate analysis of variance
qPCR: Quantitative polymerase chain reaction
sPLS: Sparse partial least squares
(s)PLS-DA: (Sparse) partial least squares-discriminant analysis
UHPLC-MS: Ultra-high-pressure liquid chromatography-mass spectrometry
VIP: Variable influence on the projection
