# Supplementary material for: Microbiome function predicts amphibian chytridiomycosis disease dynamics
Source: Microbiome. 2022 Mar 10;10:44. doi: 10.1186/s40168-021-01215-6 (PMC8908643; doi:10.1186/s40168-021-01215-6)
Supplement: Supplementary file 2 — Additional file 1. Supplementary Information. [file 40168_2021_1215_MOESM2_ESM.pdf]

Supplementary Information

Supplementary Tables

Supplementary Table 1. Summary of wild *Alytes obstetricans* populations used in this study.

Population	Elevation (m)	*Bd* first detected	*Bd* infection status
Acherito	1869	2004	Enzootic
Lhurs	1691	2009	Enzootic
Puits d'Arious	1867	2006	Enzootic
Arlet	1986	2005	Epizootic

Supplementary Table 2. Tukey's multiple comparisons of log10(GE+1) of Pyrenean midwife toad populations. Significant adjusted *p*-values show in bold.

Comparison	Acherito	Lhurs	Puits
**Acherito**	x	x	x
**Lhurs**	0.886	x	x
**Puits**	0.803	0.998	x
**Arlet**	**<0.001**	**<0.001**	**<0.001**

Supplementary Data

Supplementary Data 1. Discriminatory bacterial taxa in enzootic and epizootic populations identified by sPLS-DA.

Supplementary Data 2. Discriminatory abundant fungal taxa in enzootic and epizootic populations identified by sPLS-DA.

Supplementary Data 3. Differentially abundant bacterial taxa in enzootic and epizootic populations identified by ALDEx2.

Supplementary Data 4. Differentially abundant fungal taxa in enzootic and epizootic populations identified by ALDEx2.

Supplementary Data 5. sPLS-DA loadings for bacterial KOs from wild populations.

Supplementary Data 6. Differentially abundant bacterial KO genes in epizootic and enzootic populations identified by ALDEx2.

Supplementary Data 7. Differentially abundant metabolite features identified by univariate method (Wilcoxon test ($q < 0.05$) and log2 fold change >1.5).

Supplementary Data 8. Differentially abundant metabolite features identified by multivariate analysis (PLS-DA) and significant from Wilcoxon test ($q < 0.05$) and log2 fold change >1.5.

Supplementary Data 9. Metabolite annotations from field DIABLO results.

Supplementary Data 10. Network statistics from sPLS regression for bacteria-metabolite interactions.

Supplementary Data 11. Network statistics from sPLS regression for fungi-metabolite interactions.

Supplementary Data 12. Discriminatory bacterial taxa based on treatment on day 30 of the experiment identified by sPLS-DA.

Supplementary Data 13. Discriminatory fungal taxa based on treatment on day 30 of the experiment identified by sPLS-DA.

Supplementary Data 14. Differentially abundant bacterial taxa based on treatment on day 30 of the experiment identified by ALDEx2.

Supplementary Data 15. Differentially abundant fungal taxa based on treatment on day 30 of the experiment identified by ALDEx2.

Supplementary Data 16. Differentially abundant bacterial taxa based on treatment on day 60 of the experiment identified by ALDEx2.

Supplementary Data 17. Discriminatory bacterial KOs based on treatment on day 30 of the experiment identified by sPLS-DA.

Supplementary Data 18. Differentially abundant bacterial KOs based on treatment on day 30 of the experiment identified by ALDEx2.

Supplementary Data 19. Metabolite differences in control and exposed animals on day 30 of the experiment identified by Wilcoxon test ($q < 0.05$) and log2 fold change >1.5.

Supplementary Data 20. Metabolite differences in control and exposed animals on day 30 of the experiment with VIP > 2, log2 fold change > 1.5 and adjusted *p* value < 0.05.

Supplementary Data 21. Metabolite annotation from lab DIABLO results.

Supplementary Data 22. Shared discriminatory features from the lab and field.

Supplementary Figures

SI Figure 1. Map of study sites. Map generated using ArcGIS version 10.0 (http://www.esri.com/software/arcgis) with the World Imagery Basemap. Source: Esri, DigitalGlobe, GeoEye, Earthstar, Geographics, CNES/Airbus DS, USDA, USGS, AeroGRID, IGN, the GIS User Community.

SI Figure 2. Boxplot of intensity for second ion of putative indole-3-carboxaldeyde (*m/z* 144.04606, RT 8.73 min). Sample sizes: Acherito n=14, Lhurs n=14, Puits n=14, Arlet n=14.

SI Figure 3. Skin bacteria-metabolome interactions distinguishes wild *Bd* disease dynamics. Relevance networks produced by sPLS regression. **a)** bacterial subnetwork A, indicative of epizootic dynamics showing associations between bacterial ASVs and metabolite features **b)** bacterial subnetwork B. Metabolite features and ASVs are coloured according to identification from univariate analyses (metabolites: Wilcoxon test $q < 0.05$, log2 fold change > 1.5, ASVs: sPLS-DA analysis or ALDEx2). Sample sizes: Acherito n=14, Lhurs n=14, Puits n=14, Arlet n=14.

SI Figure 4. Skin fungi-metabolome interactions distinguishes wild *Bd* disease dynamics. Relevance networks produced by sPLS regression. **a)** fungal subnetwork A showing positive associations between epizootic OTUs and epizootic metabolite features. **b)** subnetwork B. Metabolite features and OTUs are coloured according to identification from univariate analyses (metabolites: Wilcoxon test $q < 0.05$, log2 fold change > 1.5, OTUs: sPLS-DA analysis or ALDEx2). Sample sizes: Acherito n=11, Lhurs n=6, Puits n=8, Arlet n=10.

SI Figure 5. *Bd* infection intensity in the *Bd* exposed treatment group over the course of the experiment. Sample sizes: Control=20, *Bd* exposed=20.

SI Figure 6. Trends in ASVs abundance for taxa that were discriminatory in both the laboratory and field studies. Column 1: CLR transformed abundance of ASVs for each time point of the experiment. Column 2: Spearman's correlation between CLR transformed ASV abundance and log 10 GE +1 on day 30 of the experiment. Column 3: CLR transformed abundance in wild populations. ASVs plotted are **a)** ASV17_*Stenotrophomonas* **b)** ASV45_Comamonadaceae and **c**) ASV6_*Sphingobacterium*.

SI FIGURE 1

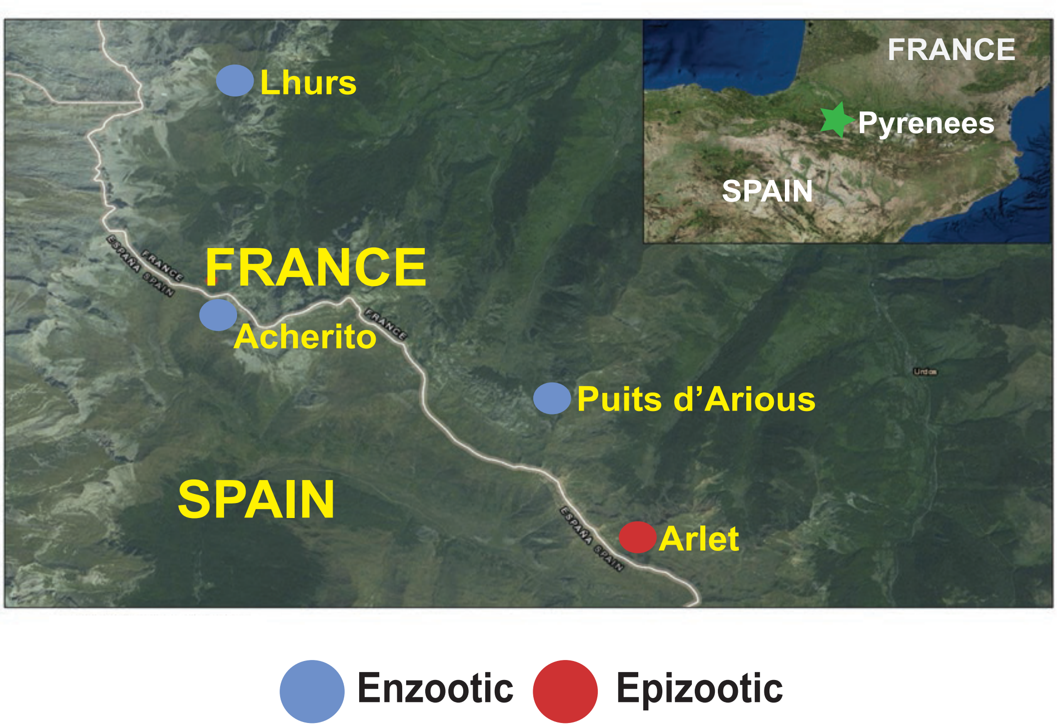
Lhurs
FRANCE
Acherito
Puits d'Arious
SPAIN
Arlet
FRANCE
Pyrenees
SPAIN
Enzootic
Epizootic

SI FIGURE 2

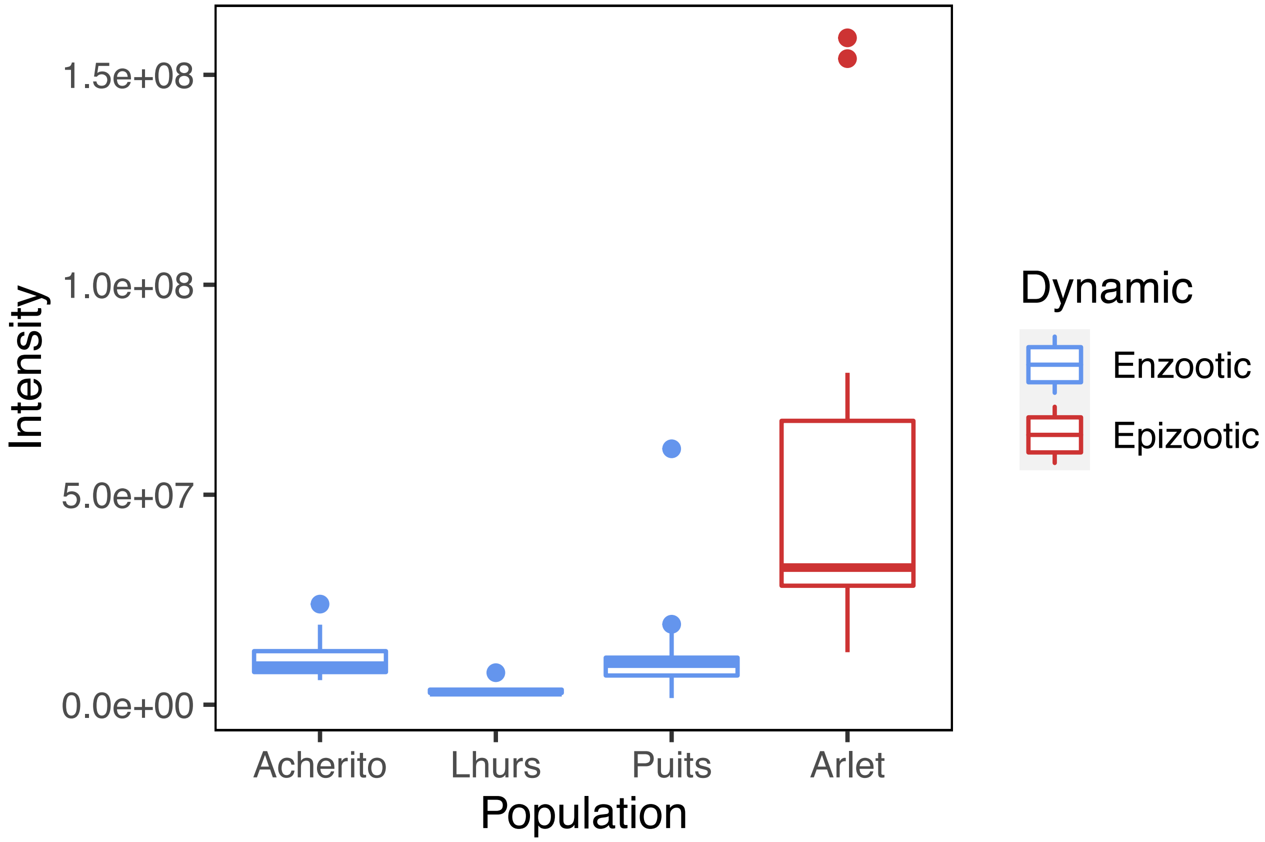
1.5e+08
1.0e+08
5.0e+07
0.0e+00
Intensity
Acherito
Lhurs
Puits
Arlet
Population
Dynamic
Enzootic
Epizootic

SI FIGURE 3

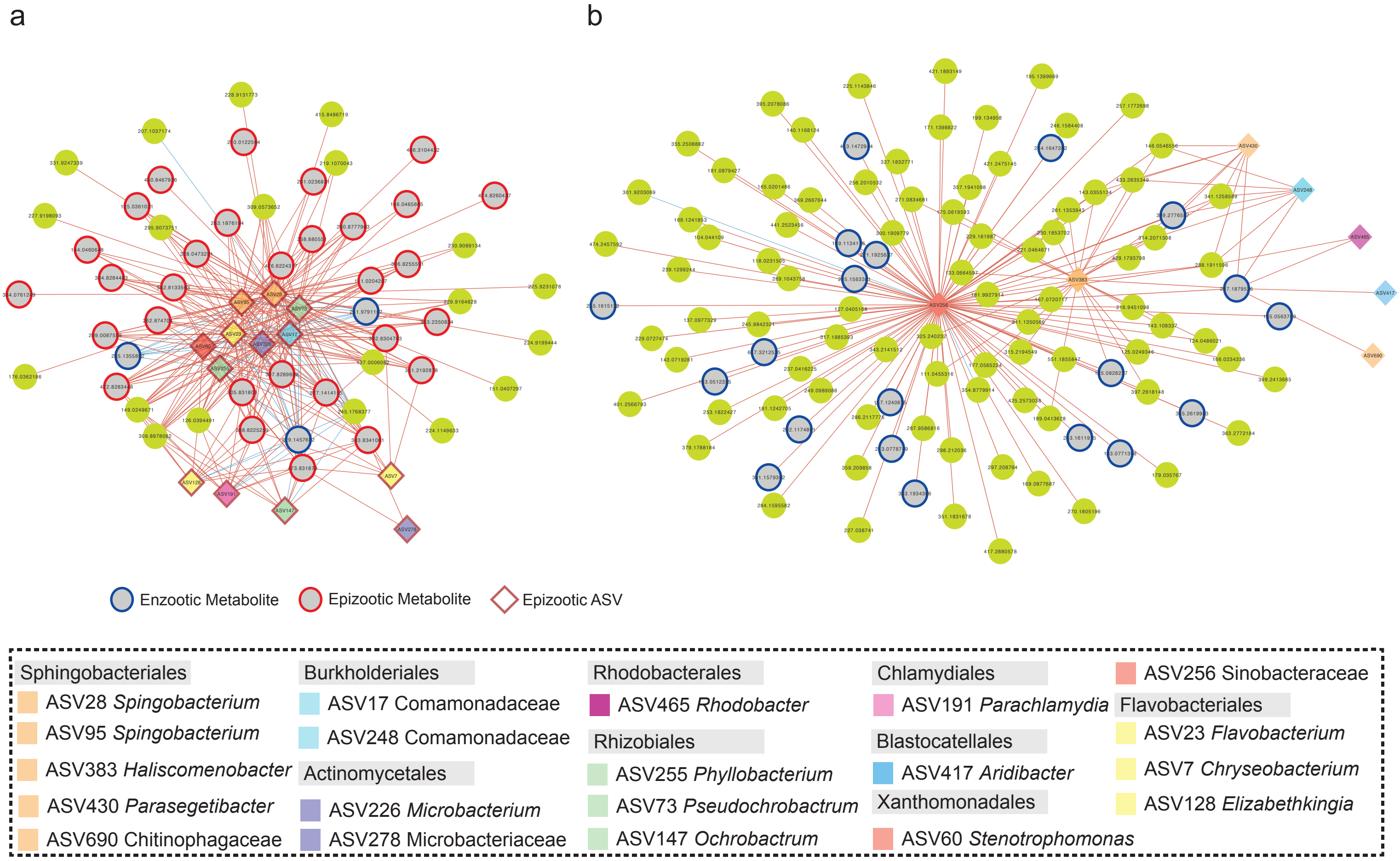

SI FIGURE 4

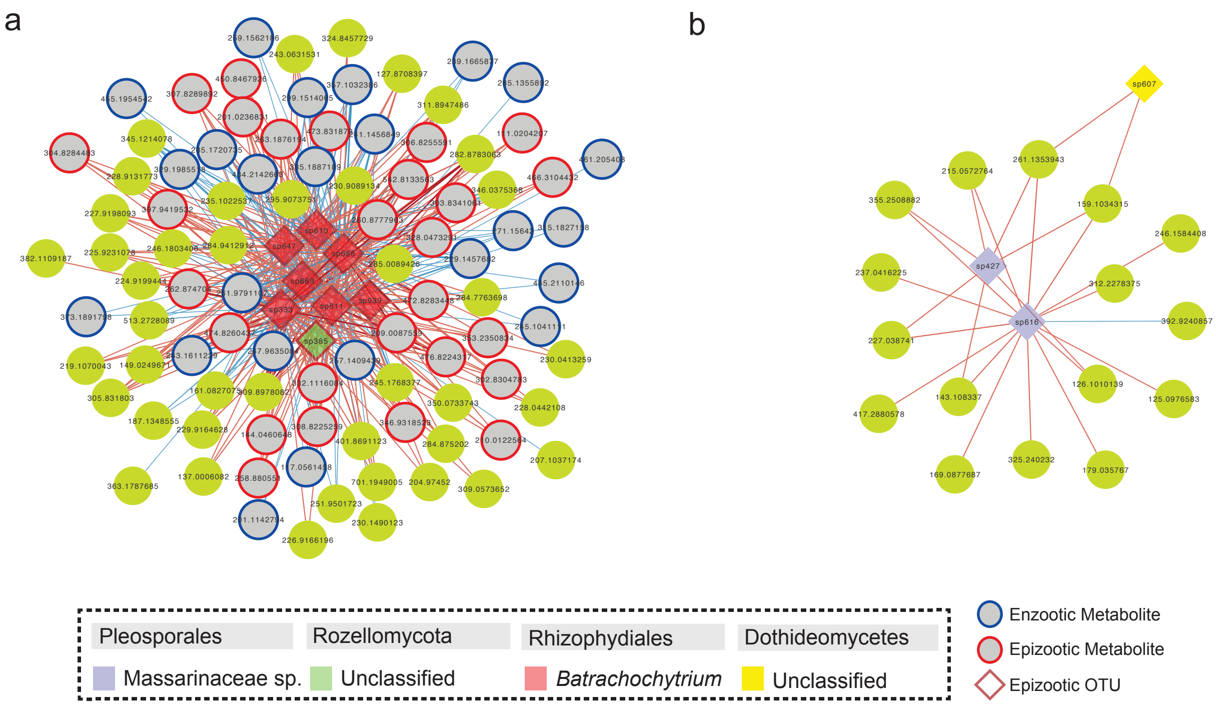

SI FIGURE 5

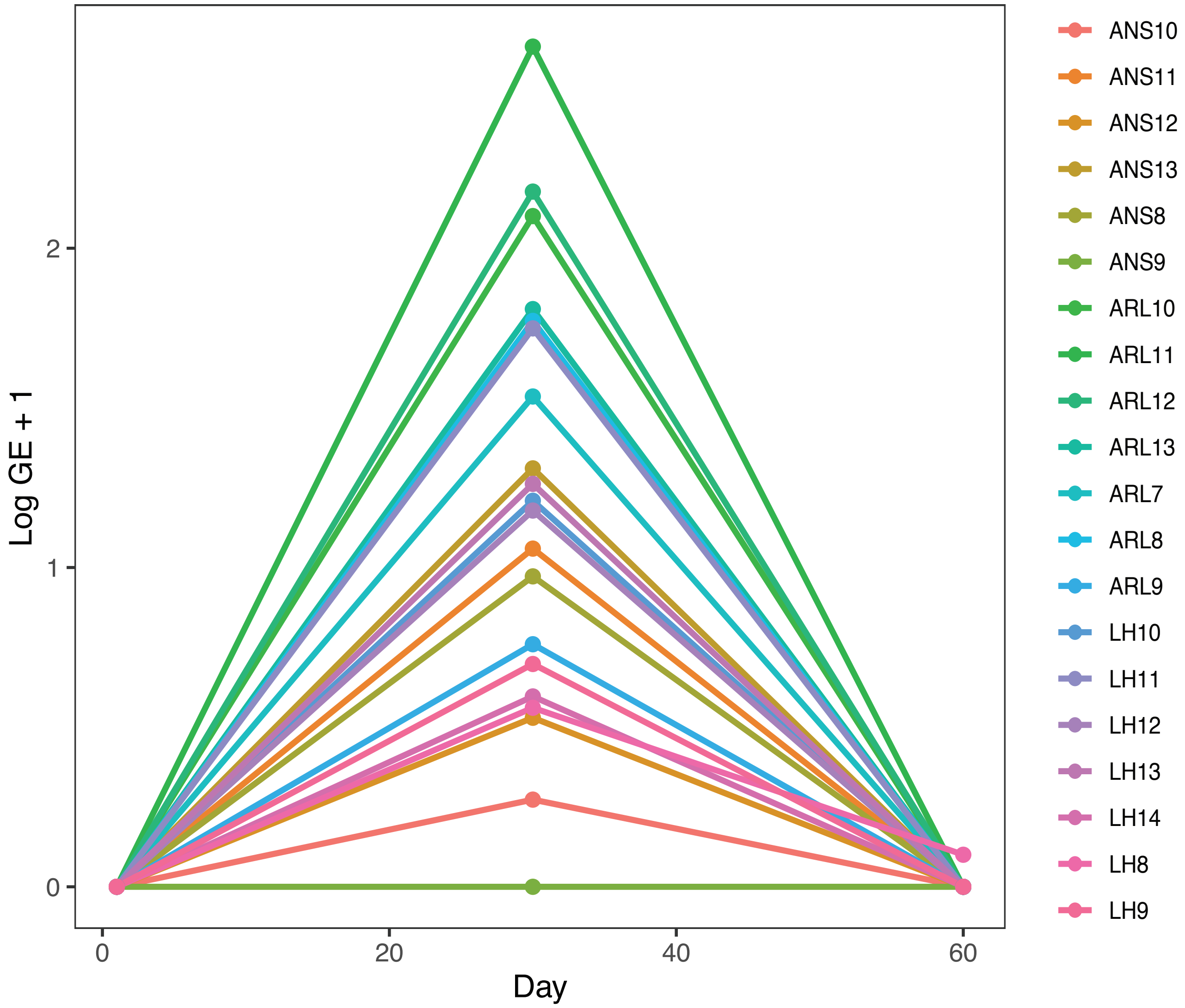

Log GE + 1
0
1
2
Day
0
20
40
60
ANS10
ANS11
ANS12
ANS13
ANS8
ANS9
ARL10
ARL11
ARL12
ARL13
ARL7
ARL8
ARL9
LH10
LH11
LH12
LH13
LH14
LH8
LH9

SI FIGURE 6

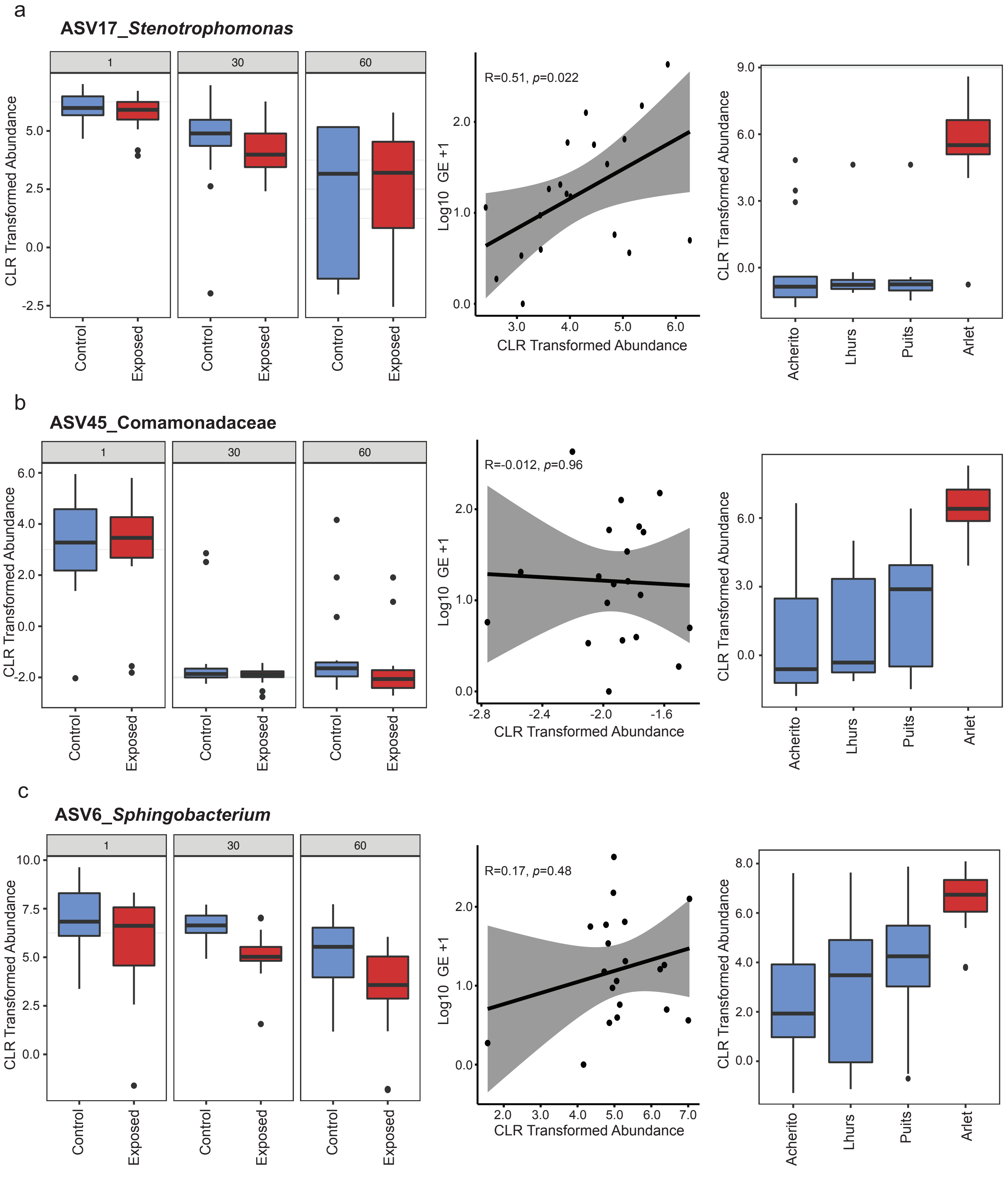
a
ASV17_Stenotrophomonas
1
30
60
CLR Transformed Abundance
Control
Exposed
R=0.51, p=0.022
Log10 GE +1
CLR Transformed Abundance
Acherito
Lhurs
Puits
Arlet
b
ASV45_Comamonadaceae
R=-0.012, p=0.96
c
ASV6_Sphingobacterium
R=0.17, p=0.48
