# Supplementary material for: Microbiome function predicts amphibian chytridiomycosis disease dynamics
Source: Microbiome. 2022 Mar 10;10:44. doi: 10.1186/s40168-021-01215-6 (PMC8908643; doi:10.1186/s40168-021-01215-6)
Supplement: Supplementary file 4 — Additional file 3. Supplementary Methods. [file 40168_2021_1215_MOESM4_ESM.docx]

**Supplementary Methods**

**Metabolite collection**

To collect metabolites from solution, 10ml of the bath solution was passed through a combination of two solid phase extraction (SPE) cartridges - a 500mg 3ml Discovery DSC-18 cartridge (Sigma Aldrich, Missouri, USA) and a 100mg 1ml ISOLUTE ENV+ cartridge (Biotage, Uppsala, Sweden) connected by an SPE tube adaptor. SPE cartridges were pre-conditioned with 5ml of methanol (MeOH) and rinsed with 5ml of sterile water immediately prior to sample collection. The liquid sample was applied at consistent flow rate of one to two drops per second to ensure optimal metabolite retention. After sample application, the SPE cartridges were rinsed with 1ml of sterile water to aid in contaminant removal. Both metabolome/microbiome swabs and SPE cartridges were placed immediately on dry ice before being transferred to a -80°C freezer.

**16s Metabarcoding**

PCR conditions consisted of a denaturing step of 95°C for 15 min, followed by 28 cycles of 95°C for 20s, 50°C for 60s, 72°C for 60s and a final extension step of 72°C for 10 min. Each PCR including a negative water control was performed in triplicate. Amplicons were visualized on a 2% agarose gel and pooled yielding a final per sample volume of 24μL. Pooled amplicon DNA was purified using an Ampure XP PCR purification kit (Beckman Coulter, California, USA). Following purification, 1μL of each combined sample was pooled into a preliminary library and the concentration was determined using Qubit fluorometric quantification (Life Technologies, California, USA). Amplicon quality and incidence of primer dimer was assessed using an Agilent 2200 TapeStation system (Agilent Technologies, California, USA). A titration run of 300 sequencing cycles was performed on a MiSeq instrument (Illumina, California, USA) to quantify the number of reads yielded per sample from the preliminary library. An equimolar concentration of each sample was then pooled into a final composite library based on the index representation from the titration run and subsequently sequenced on a 500 cycle MiSeq run with a 250 bp paired-end strategy.

**ITS2 Metabarcoding**

PCR conditions consisted of a denaturing step of 95°C for 2 min, followed by 35 cycles of 95°C for 20s, 50°C for 20s, 72°C for 5 min and a final extension step of 72°C for 5 min. Each PCR plate included a negative swab control and negative water control, and was performed in duplicate. Amplicons were visualized on a 1.5% agarose gel and pooled yielding a final per sample volume of 50μL. Pooled amplicon DNA was purified using AMPure XP bead clean-up (Beckman Coulter, California, USA). Qubit fluorometric quantification (Life Technologies, California, USA) was used to determine the concentration of each purified sample, which were equimolar pooled to create the final library sample. This pooled sample was run on an Agilent 2200 TapeStation system (Agilent Technologies, California, USA) to assess amplicon distribution and presence of primer dimer. The sample underwent 300bp

paired-end sequencing using V3 chemistry on an Illumina MiSeq platform.

**Bacterial microbiome analysis**

We used the R package DADA2^1^ to process raw 16S sequence data. Reads were quality scored and trimmed at the first appearance of a base with a quality score of two or lower. Forward reads are expected to be of higher quality so forward reads of trimmed length shorter than 240 bases and reverse reads of trimmed length 160 bases or fewer were excluded from our dataset. Reads that contained any non-assigned bases (N) were also excluded, as were reads with an expected error rate higher than two. Any reads that matched the PhiX sequencing standard genome were also removed at the read quality screening stage.

Amplicon sequence variants (ASVs) present in our sequence dataset were computed and paired reads were merged into single consensus reads. Chimeric sequences were removed from the dataset. We assigned our ASVs to taxonomic groupings using the naïve Bayesian classifier algorithm^2^ with the RDP training set (version 16)^3^. Reads assigned as cyanobacteria/chloroplast were removed.

**Fungal mycobiome analysis**

Forward and reverse reads were assigned to samples according to dual index combinations and were paired using Paired-End reAd mergeR (PEAR)^4^. Paired-end reads were trimmed by per-base quality score using MOTHUR^5^ and reads shorter than 50bp or containing ambiguous base calls were removed. UCHIME^6^ was used to identify and remove chimeric sequences, and remaining sequences were clustered into Operational Taxonomic Units (OTUs) based on 97% similarity using Cd-hit^7^. The most abundant sequence in each OTU was used for BLASTn searches against the User-friendly Nordic ITS Ectomycorrhiza (UNITE) database^8^. Unidentified sequences or those belonging to kingdoms other than “fungi” were removed, as were fungal sequences with BLASTn search result e-values >e-20 or identity <85%.

**UHPLC-MS metabolomics and spectral processing**

For metabolite extraction, the SPE columns were eluted with 800μL MeOH through the ENV+ column onto the C18 column, which was then eluted with an additional 400 μL MeOH. The combined eluate was dried under nitrogen and stored at -80°C until mass spectrometry (MS) analysis. The samples were analysed in a controlled randomised order by ultra high pressure liquid chromatography-mass spectrometry (UHPLC-MS).

For MS analysis, the samples were each taken up in 25μL of 10% MeOH and 7μL were removed from each sample to create a pooled intrastudy QC sample. The samples were analysed in a controlled randomised order by ultra high pressure liquid chromatography-mass spectrometry (UHPLC-MS) on a Thermo Scientific Q Exactive mass spectrometer attached to a Thermo Dionex Ultimate 3000 RS system, equipped with a Thermo Hypersil Gold AQ column (100 x 1 mm, 1.9μL particles). Solvents were (A) 0.1% formic acid in water and (B) 0.1% formic acid in MeOH. Liquid chromatography was performed at 40°C over 19 min at a flow rate of 80μL per minute, with 4μL injections per sample and MS measurements started at 0.01 min, with the post-column flow between 0.4 and 0.7 min directed towards waste. The gradient was started at 100% A, ramped to 25% B from 0.4 min to 1.5 min, then over 6 min to 100% B, held for 5 min, then ramped back down to 100% A over 0.1 min for re-equilibration. Data were acquired in negative ion mode, at *m/z* 100-900 Da, 70,000 resolution and an automatic gain control of 3E6 ions. Four additional runs of analyses of the intrastudy QC sample were performed at higher resolution (140,000), and again in data dependent MS/MS analyses of the highest 5 signals per MS scan.

MS1 profiling data were converted into mzML using MS Convert (ProteoWizard), and an in-house XCMS/CAMERA R-script was used to create an initial raw intensity data matrix, which was imported into MatLab and processed using the Birmingham SimStitch pipeline (version 3.1). Signal filtering was applied to retain only those peaks with an intensity greater than twice the extraction blank and an 80% sample filter (i.e. signals have to be found in at least 80 % of samples), applied per group, to generate a sample filtered matrix. The dataset was normalised using the probabilistic quotient normalisation (PQN), missing values were imputed using a k-nearest neighbour algorithm (knn, k=5) and for multivariate statistical analyses, the data were transformed using a generalised logarithm (g-log)^9,10^. Putative metabolite annotations were created using the in-house software MIPack^11^, matching *m/z* values against the KEGG database (www.genome.jp/kegg/pathway.html). Higher-resolution and LC-MS/MS runs acquired for QC samples directly before the main sequence served to check the plausibility of annotations for signals of interest. Fragmentation data for indole-3-carboxaldehyde (a known anti-*Bd* metabolite of interest) was directly compared to an authentic standard (acquired by direct infusion MS).

**References**

1. Callahan, B. J. *et al.* DADA2: High-resolution sample inference from Illumina amplicon data. *Nat Methods* **13**, 581–583 (2016).

2. Wang, Q., Garrity, G. M., Tiedje, J. M. & Cole, J. R. Naive bayesian classifier for rapid assignment of rRNA sequences into the new bacterial taxonomy. *Applied and Environmental Microbiology* **73**, 5261–5267 (2007).

3. Cole, J. R. *et al.* The Ribosomal Database Project: improved alignments and new tools for rRNA analysis. *Nucleic Acids Research* **37**, D141–D145 (2009).

4. Zhang, J., Kobert, K., Flouri, T. & Stamatakis, A. PEAR: a fast and accurate Illumina Paired-End reAd mergeR. *Bioinformatics* **30**, 614–620 (2014).

5. Schloss, P. D. *et al.* Introducing mothur: open-source, platform-independent, community-supported software for describing and comparing microbial communities. *Applied and Environmental Microbiology* **75**, 7537–7541 (2009).

6. Edgar, R. C., Haas, B. J., Clemente, J. C., Quince, C. & Knight, R. UCHIME improves sensitivity and speed of chimera detection. *Bioinformatics* **27**, 2194–2200 (2011).

7. Li, W. & Godzik, A. Cd-hit: a fast program for clustering and comparing large sets of protein or nucleotide sequences. *Bioinformatics* **22**, 1658–1659 (2006).

8. Kõljalg, U. *et al.* UNITE: a database providing web-based methods for the molecular identification of ectomycorrhizal fungi. *New Phytologist* **166**, 1063–1068 (2005).

9. Parsons, H. M., Ludwig, C., Günther, U. L. & Viant, M. R. Improved classification accuracy in 1- and 2-dimensional NMR metabolomics data using the variance stabilising generalised logarithm transformation. *BMC Bioinformatics* **8**, 234 (2007).

10. Di Guida, R. *et al.* Non-targeted UHPLC-MS metabolomic data processing methods: a comparative investigation of normalisation, missing value imputation, transformation and scaling. *Metabolomics* **12**, 93 (2016).

11. Weber, R. J. M. & Viant, M. R. MI-Pack: Increased confidence of metabolite identification in mass spectra by integrating accurate masses and metabolic pathways. *Chemometrics and Intelligent Laboratory Systems* **104**, 75–82 (2010).
